# Expanding the Boundaries of Minimally Invasive Cardiac Surgery: Initial Experience with Multivalve Procedures

**DOI:** 10.3390/jcm15124424

**Published:** 2026-06-08

**Authors:** Wojciech Karolak, Aleksandra Stańska, Igor Tomczyk, Andrzej Klapkowski

**Affiliations:** 1 Department of Cardiac Surgery, Faculty of Medicine, Medical University of Gdańsk, 80-210 Gdansk, Poland; itomczyk@uck.gda.pl (I.T.); a.klapkowski@gmail.com (A.K.); 2 Division of Quality of Life Research, Department of Psychology, Faculty of Health Sciences, Medical University of Gdańsk, 80-210 Gdansk, Poland; astanska@gumed.edu.pl

**Keywords:** minimally invasive cardiac surgery, multivalve surgery, double-valve surgery, aortic valve replacement, mitral valve surgery, minithoracotomy, cardiopulmonary bypass

## Abstract

**Background/Objectives**: Minimally invasive valve surgery via right minithoracotomy is well established for isolated aortic and mitral procedures, but its application to multivalve operations remains uncommon and clinical data are scarce. We report our initial single-center experience with minimally invasive multivalve surgery—defined as a small skin incision without rib spreading or internal mammary artery dissection—in patients with combined aortic and mitral disease. **Methods**: We retrospectively analyzed 10 consecutive patients who underwent minimally invasive multivalve cardiac surgery at our institution. All operations were performed through a 5–7 cm right minithoracotomy in the third or fourth intercostal space, with femoral cannulation for cardiopulmonary bypass (CPB). Nine patients underwent a double-valve procedure (aortic and mitral) and one a triple-valve procedure (aortic, mitral, and tricuspid). Operative variables, perioperative complications, and early echocardiographic outcomes were assessed. **Results**: The mean age of patients was 69.8 ± 5.2 years and 60% were female. Mean CPB and aortic cross-clamp times were 197.6 ± 48.3 min and 148.1 ± 34.7 min, respectively. All procedures were completed via the minimally invasive approach, with no conversion to sternotomy and no in-hospital deaths. No rethoracotomies, wound infections, or peripheral vascular complications occurred. Postoperative atrial fibrillation, observed in five patients (50%), was the most common complication. Early echocardiography showed good valve function in nine patients (90%); one had a moderate aortic paravalvular leak managed conservatively. **Conclusions**: In a center with established experience in single-valve minimally invasive surgery, multivalve procedures can be safely extended to a right minithoracotomy approach, with low perioperative morbidity and no early mortality despite operative times reflecting the early learning curve.

## 1. Introduction

Valvular heart disease is an increasing public health burden worldwide, and surgical valve repair or replacement remains the standard of care for symptomatic severe disease, particularly when transcatheter approaches are anatomically unsuitable or contraindicated. For decades, the median sternotomy has served as the standard surgical access for most cardiac valve operations. While this approach offers excellent exposure and reproducibility, it is associated with substantial perioperative morbidity, prolonged recovery, persistent postoperative pain, and the potential for serious sternal wound complications [1].

Over the past two decades, minimally invasive valve operations—performed through a small right anterolateral or axillary minithoracotomy, with or without endoscopic or fully endoscopic visualization—have emerged as effective and safe alternatives to full sternotomy [1,2,3]. These approaches are associated with reduced blood loss and pain, lower morbidity, faster recovery, and shorter intensive care unit and hospital stays, while achieving comparable durability of valve repair or replacement [2,3]. Patient demand for less invasive surgical options has consequently grown, particularly among younger and active patients.

Despite this progress, the application of minimally invasive techniques to combined multivalve procedures—most commonly the simultaneous treatment of aortic and mitral valve disease—remains uncommon. Multivalve operations are technically more demanding, involve longer cardiopulmonary bypass (CPB) and aortic cross-clamp times, and require precise sequencing of the aortic and mitral procedures through limited access. Published data on truly minimally invasive multivalve surgery—defined here as a procedure performed through a small skin incision without rib spreading or internal mammary artery dissection—are largely limited to single cases or small case series [4,5,6,7,8,9,10], with only a few high-volume centers reporting larger experiences [11].

At our institution, minimally invasive valve operations have been the standard of care for many years. Building on substantial experience with isolated aortic and mitral valve surgery through this approach, we extended its benefits to patients with combined multivalve disease. The aim of this study was to describe our initial single-center experience with minimally invasive multivalve surgery and to evaluate its feasibility, safety, and early outcomes.

## 2. Materials and Methods

### 2.1. Study Design and Patient Population

This was a retrospective, single-center observational study of consecutive patients who underwent minimally invasive multivalve cardiac surgery at the Department of Cardiac Surgery, Medical University of Gdańsk, Poland. Patients were eligible for inclusion if they underwent combined aortic and mitral valve surgery (with or without concomitant tricuspid valve repair) performed through a right minithoracotomy. Patients undergoing emergency operations, redo cardiac surgery, or multivalve procedures for active infective endocarditis were not included in the present cohort. All patients were assessed preoperatively by the institutional Heart Team. All procedures performed in this study involving human participants were in accordance with institutional and national research standards. The study was conducted in accordance with the ethical principles outlined in the Declaration of Helsinki. Ethics approval was waived given the retrospective nature of the analysis and the use of anonymized clinical data collected during routine perioperative care. The requirement for separate written informed consent for study participation was also waived; all patients had previously signed standard institutional consent for the surgical procedure.

All ten patients in this initial series were operated on by two experienced surgeons (A.K. and W.K.) who perform minimally invasive surgery. Aside from the exclusion criteria mentioned, other patients who had combined multivalve procedures were operated on by non-minimally invasive surgeons and thus had sternotomy. Patient selection was solely based on preoperative anatomical CT suitability for combined valve procedure (AVR/MVR) based on the trans-axillary AVR suitability described previously by Marco Di Eusanio. Eight patients underwent combined aortic and mitral valve replacement, two underwent aortic valve replacement with mitral valve repair, and one of the patients receiving combined aortic and mitral valve replacement additionally underwent tricuspid valve annuloplasty.

### 2.2. Surgical Technique

All operations were performed under general anesthesia with single-lumen or double-lumen endotracheal intubation according to surgeon preference. Patients were positioned supine with the right chest elevated by 30 degrees and the right upper extremity placed overhead. Defibrillation pads were applied to the chest wall before draping.

The main minithoracotomy incision (5–7 cm) was made in the third or fourth intercostal space at the anterior axillary line. The optimal level was selected on the basis of preoperative chest computed tomography (CT) angiography, which was performed in all patients to evaluate the relationship of the heart and great vessels to the chest wall as well as the suitability of the femoral vessels for cannulation. Soft tissue retractors were used without rib spreading, and no portion of the rib or the internal mammary artery was divided. Additional small incisions were placed for the Chitwood transthoracic aortic clamp (one interspace above the main incision) and, when preferred by the surgeon, for an endoscope (within the same interspace as the main incision). All 10 procedures were performed using video-assisted direct-vision.

Cardiopulmonary bypass was established through femoral cannulation, performed either percutaneously or by surgical cutdown according to the surgeon’s experience and preference. Operations were conducted under normothermia or mild hypothermia (34 °C). Cardioplegia was delivered using either HTK Bretschneider solution (eight patients) or del Nido solution (two patients), administered antegrade through an aortic root needle, with selective direct ostial delivery if required. After aortic cross-clamping and cardioplegia delivery, the diseased aortic valve was excised first; the mitral valve was then addressed via a left atriotomy. Once the mitral procedure was completed, the left atrium was closed and the aortic valve replacement was performed. The aorta was closed in a standard fashion.

Before declamping the aorta, temporary pacing wires were sutured onto the diaphragmatic surface of the right ventricle. Carbon dioxide was continuously insufflated into the pleural cavity throughout the procedure to facilitate de-airing. Following standard de-airing maneuvers, the patient was weaned from CPB, and prosthetic and repair valve function was assessed using transesophageal echocardiography (TEE). Hemostasis was verified, drains were placed, and the chest was closed in a standard fashion.

### 2.3. Data Collection and Outcomes

Demographic, clinical, operative, and outcome data were extracted from the institutional electronic medical record. Operative variables included CPB time, aortic cross-clamp time, cardioplegia strategy, and conversion to sternotomy. Perioperative outcomes assessed included in-hospital mortality, rethoracotomy for bleeding, surgical wound infection, peripheral vascular complications related to femoral cannulation, postoperative atrial fibrillation, and other major complications. Early postoperative TEE or transthoracic echocardiography (TTE) was performed prior to discharge in all patients to evaluate prosthetic and repair valve function and left ventricular ejection fraction (LVEF). Follow-up echocardiographic data were collected at routine postoperative outpatient visits when available.

### 2.4. Statistical Analysis

Given the descriptive nature of this initial case series, no formal hypothesis testing was performed. Continuous variables are presented as mean ± standard deviation (SD) with range; categorical variables are presented as counts and percentages.

The authors used generative artificial intelligence tools (Claude and Gemini) exclusively for language editing, grammar correction, improvement of readability, and preparation of the cover letter. The AI tools were not used for data collection, data analysis, statistical calculations, interpretation of results, generation of scientific conclusions, or manuscript content creation. All outputs were carefully reviewed, verified, and approved by the authors, who take full responsibility for the final content of the manuscript.

## 3. Results

### 3.1. Patient Characteristics

The study cohort comprised 10 consecutive patients undergoing minimally invasive multivalve cardiac surgery. The mean age was 69.8 ± 5.2 years (range, 60–75), with a predominance of female patients (60%). All procedures were performed as primary interventions; there were no cases of prior cardiac surgery, and no patient had active infective endocarditis. Baseline characteristics and procedural data are summarized in Table 1. Pulmonary hypertension was defined via tricuspid regurgitation (TR) and estimated by the right ventricular systolic pressure (RVSP)—with high probability being RVSP > 45 mmHg.

### 3.2. Operative Data

Aortic valve replacement was performed in all cases using biological prostheses. Mitral valve intervention consisted of replacement in eight patients (80%) and repair in two patients (20%); mitral repairs included annuloplasty rings and the implantation of artificial chordae. One patient (10%) additionally underwent tricuspid valve repair, constituting a triple-valve procedure. The mean CPB time was 197.6 ± 48.3 min (range, 125–280), and the mean aortic cross-clamp time was 148.1 ± 34.7 min (range, 96–210). HTK Bretschneider cardioplegia was used in eight patients (80%) and del Nido cardioplegia in two patients (20%). Patient specific valve lesions are described in Table 2. Only one patient (patient 2) operated on at the beginning of the minimally invasive combined valve program, who was deemed to be a mitral valve repair candidate, underwent replacement to err on the side of caution. Only one patient demonstrated severe TR and thus underwent tricuspid valve repair.

### 3.3. Perioperative Outcomes

All procedures were successfully completed via the minimally invasive approach, with no conversion to full sternotomy. There were no in-hospital deaths. No rethoracotomies for bleeding, surgical wound infections, or peripheral vascular complications were observed. Postoperative atrial fibrillation, occurring in five patients (50%), was the most common complication and was managed medically in all cases.

### 3.4. Echocardiographic Outcomes

Early postoperative echocardiography (3 months) demonstrated good prosthetic and repair function in nine patients (90%). One patient (10%) presented with a moderate paravalvular leak of the aortic prosthesis. The patient remained asymptomatic, was placed under close clinical and echocardiographic surveillance, and required neither surgical nor percutaneous reintervention during the available follow-up. Left ventricular ejection fraction ranged from 40% to 60% (mean approximately 53%). Where available, follow-up echocardiography confirmed stable prosthetic and repair function.

## 4. Discussion

This initial single-center series demonstrates that minimally invasive multivalve cardiac surgery—performed through a small right minithoracotomy without rib spreading or internal mammary artery dissection—can be carried out safely and effectively in selected patients with combined aortic and mitral valve disease. All procedures were completed without conversion to sternotomy, there were no in-hospital deaths, and major non-cardiac complications were absent. The most common complication was postoperative atrial fibrillation, which is consistent with the published experience after open and minimally invasive valve surgery.

Multivalve operations are traditionally performed through a median sternotomy, which provides excellent exposure of the thoracic cavity and makes these complex procedures safe and reproducible. At our institution, around 15–20 combined AVR/MVR procedures are performed yearly. However, the sternotomy approach carries several drawbacks, including prolonged recovery, persistent postoperative pain, and the potential for serious sternal wound complications [1]. As a result, an increasing number of patients prefer to avoid sternotomy in favor of less invasive options, and contemporary surgical practice has progressively expanded minimally invasive techniques into more complex anatomical scenarios [2,3].

At our institution, minimally invasive valvular procedures—including both direct-vision and endoscopic mitral and aortic valve operations—have been performed for many years. Building on this experience, and in response to growing patient demand, we extended our minimally invasive program to include double- and triple-valve operations. As demonstrated in the present series aiming to show in-hospital and short-term (3-month) follow-up, a minimally invasive approach can be applied safely even to such complex conditions, with a very low complication rate. CPB and aortic cross-clamp times in our cohort were relatively long, which most likely reflects the early phase of our learning curve for combined multivalve procedures through a single minithoracotomy. Importantly, these prolonged operative times did not translate into a higher rate of perioperative complications or mortality.

Data on truly minimally invasive multivalve operations remain scarce. Most published reports consist of single cases or small case series of minimally invasive double-valve surgery [4,5,6,7,8]. Salvador et al. show that even triple-valve minimally invasive surgery can be combined with an ascending aorta replacement [10]. Two larger case series highlight the feasibility of minimally invasive combined procedures. Lamelas reported a substantial single-center experience with concomitant aortic and mitral valve surgery performed through a right anterior minithoracotomy—the so-called “Miami Method”—and described markedly shorter CPB and cross-clamp times than those observed in our cohort [9]. Zoni et al. described over a hundred combined minimally invasive procedures—their novelty was the fact that minimally invasive AVR was combined not just with other valve procedures. This group showed that revascularization, ablation, myectomy and others can all be combined, and in experienced centers can be addressed minimally invasively and with excellent outcomes [9]. Direct comparison between our results and those of an established high-volume program, like those of Lamelas et al. and Salvador et al., with more than two decades of experience is, however, inherently limited. Differences in case mix, surgical team experience, perfusion strategies, and the maturity of the institutional pathway around the minimally invasive program all contribute to variability in operative times. The clinically relevant observation is that, despite longer operative times during the early phase of our experience, perioperative outcomes were comparable, with no in-hospital mortality and a low rate of major complications.

The single case of moderate aortic paravalvular leak observed in our series highlights the technical demands of aortic valve replacement performed through a remote minithoracotomy in the setting of a concomitant mitral procedure. Conservative management was appropriate in this asymptomatic patient, and continued surveillance is warranted. As experience accumulates and the team progresses along the learning curve, further reductions in operative times and refinement of valve seating are expected, in line with reports from other centers [3,7,9].

### Limitations

This study has several important limitations. First, it is a retrospective, single-center, observational analysis of a small consecutive cohort of 10 patients without a comparator group, which limits the ability to draw firm conclusions about the relative safety and efficacy of the minimally invasive approach versus conventional sternotomy in this population. Second, follow-up was confined to early postoperative outcomes and the available outpatient echocardiographic studies; long-term data on valve durability, reintervention, and survival are not yet available and will be subject to future publications. Third, all procedures were performed by two experienced surgeons within an established minimally invasive valve program, and our results may therefore not be directly generalizable to lower-volume settings or to teams in the very early phase of adopting minimally invasive techniques. Fourth, although the cohort is described as consecutive, patient selection was guided by Heart Team assessment and by anatomical suitability on preoperative CT angiography, introducing inherent selection. Larger, prospective, multicenter studies with appropriate comparator groups will be required to formally establish the role of minimally invasive multivalve surgery.

## 5. Conclusions

Multivalve cardiac procedures remain complex operations that are still performed through a conventional sternotomy in most centers. Our initial experience suggests, however, that they can be carried out safely and effectively through a right minithoracotomy approach in centers with established expertise in minimally invasive single-valve surgery, with low perioperative morbidity and no early mortality despite longer operative times during the learning phase. These findings support the feasibility of minimally invasive multivalve surgery as a therapeutic option for patients seeking to avoid sternotomy and provide a basis for further prospective evaluation.

## Figures and Tables

**Table 1:** Baseline characteristics and procedural data.

Variable	Value
Number of patients	10
Age, mean ± SD (range), years	69.8 ± 5.2 (60–75)
Female sex, *n* (%)	6 (60)
Reoperation, *n* (%)	0 (0)
Infective endocarditis, *n* (%)	0 (0)
**Echocardiographic Data**	
LVEF, mean ± SD (range), %	53.4 ± 7.1 (40–60)
Pulmonary hypertension, *n* (%)	5 (50%)
**Valve Pathology Etiology**	
Aortic stenosis, *n* (%)	10 (100)
Aortic insufficiency, *n* (%)	4 (40)
Mitral stenosis, *n* (%)	5 (50)
Mitral regurgitation, *n* (%)	10 (100)
Severe tricuspid insufficiency, *n* (%)	1 (10)
**Procedures**	
Aortic valve replacement, *n* (%)	10 (100)
Mitral valve replacement, *n* (%)	8 (80)
Mitral valve repair, *n* (%)	2 (20)
Tricuspid valve repair, *n* (%)	1 (10)
**Operative data**	
CPB time, mean ± SD, min	197.6 ± 48.3
Cross-clamp time, mean ± SD, min	148.1 ± 34.7

Abbreviations: CPB, cardiopulmonary bypass; SD, standard deviation, LVEF—Left Ventricle Ejection Fraction.

**Table 2:** Patient specific valve pathology.

Patient Specific Valve Pathology	
Patient 1	Severe AS
	Severe MS and moderate MR
	Mild TR
Patient 2	Severe AS, mild AI
	Severe MR (annular dilatation)
	Mild TR
Patient 3	Severe AS
	Severe MR (flail P2)—**repaired**
	Mild TR
Patient 4	Severe AS
	Severe MR (flail P2)—**repaired**
	Mild TR
Patient 5	Severe AS, mild AI
	Severe MR (post-rheumatic)
	Moderate TR
Patient 6	Severe AS
	Moderate MS and severe MR
	Moderate TR
Patient 7	Moderate AS and AI
	Moderate MS and severe MR
	Moderate TR
Patient 8	Severe AS, mild AI
	Moderate MS and severe MR
	Mild TR
Patient 9	Severe AS
	Moderate MS and mild MR
	Moderate TR
Patient 10	Severe AS
	Severe MR (restrictive)
	Severe TR

AS—aortic stenosis; AI—aortic insufficiency; MS—mitral stenosis; MR—mitral regurgitation; TR—tricuspid regurgitation.

## Data Availability

The data presented in this study are available on reasonable request from the corresponding author. The data are not publicly available due to patient privacy restrictions.
